# Usefulness of combined androgen blockade therapy with gonadotropin-releasing hormone antagonist for bone metastatic prostate cancer with pretreatment prostate-specific antigen level ≥ 50 ng/mL

**DOI:** 10.1186/s12885-018-4541-0

**Published:** 2018-05-31

**Authors:** Takeshi Kashiwabara, Sayo Suda

**Affiliations:** Department of Urology, Saku Central Hospital, 197 Usuda, Saku, Nagano, 384-0393 Japan

**Keywords:** Bone metastasis, Combined androgen blockade, Gonadotropin-releasing hormone receptor antagonist, Prostate cancer

## Abstract

**Background:**

This study was performed to examine the usefulness of combined androgen blockade (CAB) therapy with a gonadotropin-releasing hormone (GnRH) antagonist (CAB-antagonist therapy), instead of CAB therapy with GnRH agonist (CAB-agonist therapy) against very high-risk prostate cancer (Pca).

**Methods:**

We retrospectively studied 84 Pca patients with pretreatment prostate-specific antigen (PSA) level ≥ 50 ng/mL, who were pathologically diagnosed between January 2007 and December 2016. GnRH antagonist was administered to 34 patients and GnRH agonist was administered to 50 patients. All patients received concurrent antiandrogen treatment.

The primary end point was PSA progression-free survival (PSA-PFS).

**Results:**

PSA-PFS was significantly longer for the CAB-antagonist group compared to the CAB-agonist group (log-rank test, *P* <  0.01) in Pca patients with more than six bone metastases (the extent of disease [EOD] grade 2–4). On multivariate analysis, CAB-antagonist therapy was shown to be a possible prognostic factor for PSA-PFS (adjusted hazard ratio: 0.41, 95% confidence interval: 0.16–0.90, *P* = 0.03).

**Conclusions:**

CAB-antagonist therapy may be a useful option in bone metastatic Pca patients with EOD grade 2–4.

## Background

Japan had 92,600 patients with prostate cancer (Pca) in 2016, making it the most common form of cancer among men in the country, and both the incidence and number of deaths from Pca are increasing. The 5- and 10-year survival rates of non-metastatic Pca are close to 100%. However, metastatic Pca shows 5- and 10-year survival rates of 62 and 49%, respectively, and many patients that died of Pca had advanced cancer at the time of diagnosis [1]. In 2014, 14% of patients were found to have suffered from metastatic Pca in Japan. Japan’s Clinical Practice Guidelines recommended combined androgen blockade (CAB) as the standard therapy for metastatic Pca. CAB therapy, which involves concurrent use of a gonadotropin-releasing hormone (GnRH) agonist and non-steroidal antiandrogen (CAB-agonist), is more effective than androgen deprivation therapy (ADT) alone and is recommended as the standard treatment for high-risk Pca in Japan. Patients on primary ADT in Japan were reported to have an adjusted prostate cancer-specific mortality rate less than half those in the USA. The adverse event of CAB is tolerable and the cost of CAB is acceptable for patients. Although these guidelines take into account that there is no clear evidence of the efficacy of CAB in metastatic Pca, CAB-agonist therapy is widely used in treatment of advanced or metastatic Pca throughout Japan [2]. However, it remains difficult to improve the prognosis of metastatic Pca, and an improved therapeutic modality is required.

Recently, next-generation CAB therapy, abiraterone with ADT, was reported to significantly prolong overall survival (OS) and progression-free survival (PFS) in metastatic and hormone-sensitive Pca (HSPC) compared to ADT alone. Most of the metastatic Pca patients in these studies received GnRH agonist as ADT [3, 4]. Unlike GnRH agonists, the GnRH antagonist, degarelix, neither induces a transient rise in testosterone nor aggravates the symptoms. Antiandrogen was administered concurrently in 83% of patients treated with degarelix in Japan. It is of interest to determine whether there are differences in efficacy between GnRH antagonist and GnRH agonist in CAB therapy.

Sixty-five percent of Pca patients with prostate-specific antigen (PSA) level > 50 ng/mL have metastatic disease, and optimal management for these patients is controversial [5]. Patients with PSA level > 50 ng/mL were reported to have higher risk of PSA recurrence than those with PSA level 20–50 ng/mL [6]. The baseline serum alkaline phosphatase (ALP) in patients with PSA level > 50 ng/mL is four times higher than in those with PSA level < 50 ng/mL, and high serum ALP indicated metastatic disease in patients with PSA level > 50 ng/mL [7]. PSA control after treatment was reported to be associated with improved OS. GnRH antagonist monotherapy was shown to be associated with improved PSA-PFS compared with CAB-agonist therapy [8]. Patients with PSA level ≥ 50 ng/mL were suitable for inclusion in our retrospective patient cohort study. Here, we compared the therapeutic effects of CAB using concurrent GnRH antagonist (CAB-antagonist) and CAB-agonist therapy in treatment of high-risk Pca with PSA level ≥ 50 ng/mL.

## Methods

**Fig. 1:**
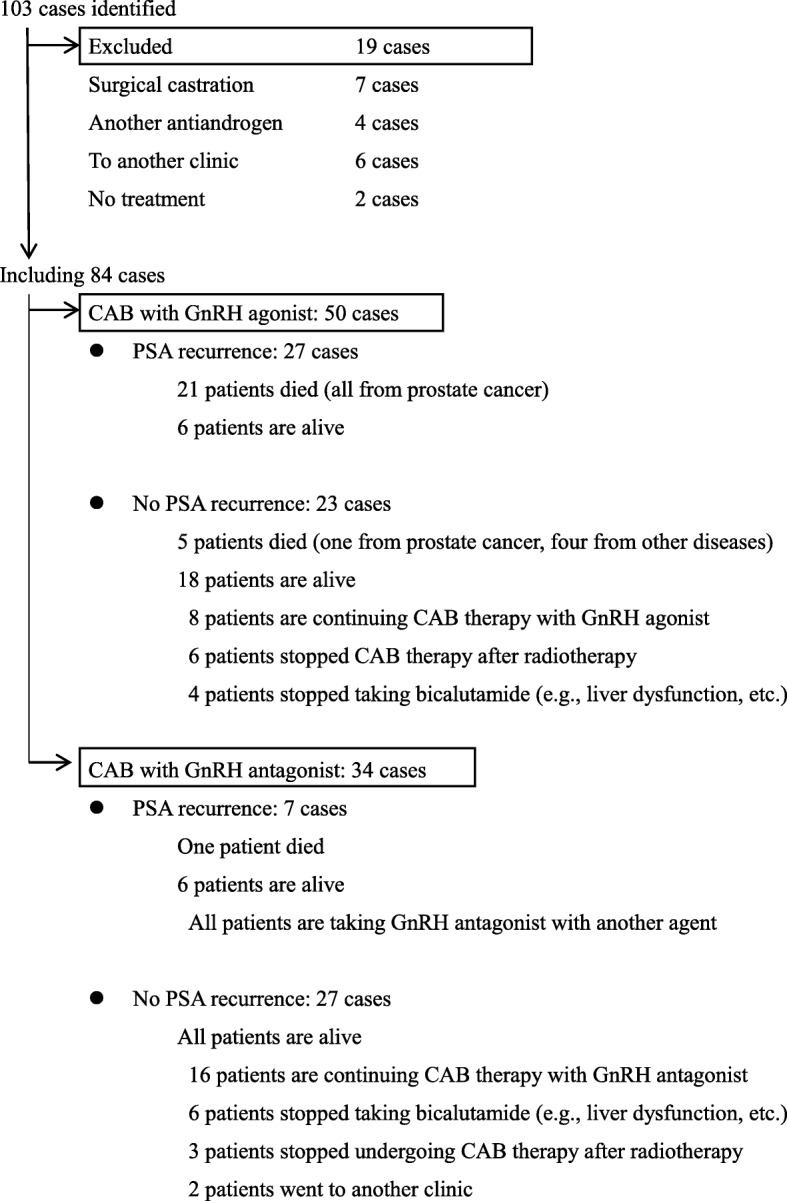
Selection and outcome of patients pathologically diagnosed with pretreatment PSA level ≥ 50 ng/mL

We identified 103 patients with a pathological diagnosis of Pca with PSA level ≥ 50 ng/mL at our hospital between January 2007 and December 2016 (Fig. 1). All cases were followed up for more than 12 weeks. GnRH antagonist (degarelix) or GnRH agonist (leuprorelin in 13 cases and goserelin in 37 cases) was administered as ADT. Oral non-steroidal antiandrogen (bicalutamide, 80 mg/daily) was begun before or concomitant with the start of ADT.

Diagnosis during the clinical phase was performed by bone scintigraphy, magnetic resonance imaging (MRI), and computed tomography (CT). The timing of PSA recurrence was defined as the day on which the PSA level increased by ≥ 25% and ≥ 2.0 ng/mL from the nadir level. If PSA did not decrease from the baseline, PSA recurrence was confirmed in the 12th week from the day on which treatment was started.

The primary end point was PSA-PFS, whereas the secondary end point was OS.

The chi-square test was used for comparison of the rates between the two groups and Wilcoxon’s rank sum test was used for comparison of the median values between the two groups. Kaplan–Meier analysis was used to estimate the differences in time to events between the CAB-antagonist group and the CAB-agonist group using the log-rank test. Prognostic factors consisted of age at the time of diagnosis, bone metastasis, Gleason score (GS), and application of CAB-antagonist therapy, and multivariate analyses were performed with Cox’s proportional hazard models. Statistical analyses were performed using SAS JMP, Version 13, and *P* <  0.05 was taken to indicate statistical significance.

This study was approved by the Institutional Review Board of Saku Central Hospital.

## Results

The study population consisted of 34 patients in the CAB-antagonist group and 50 in the CAB-agonist group. Their clinical characteristics and observation periods are shown in Table 1. Pathologically, there were 24 cases (71%) in the CAB-antagonist group and 40 (80%) in the CAB-agonist group with GS ≥ 8. The difference between the two groups was not significant (*P* = 0.19). In the total population, Seventy patients (83%) had primary tumor category ≥ T3, 57 patients (69%) had metastatic Pca, 44 (54%) had bone metastasis, and 46 (56%) had lymph node metastasis. Thirty-two patients (40%) had concurrent bone metastasis and lymph node metastasis, 11 (14%) had bone metastasis alone, and 13 (17%) had only lymph node metastasis. Among the cases with bone metastasis, 13 (72%) in the CAB-antagonist group and 19 (73%) in the CAB-agonist group were treated with denosumab or zoledronic acid, respectively; the difference between the two groups was not significant. Thirty-five (69%) of 52 patients with PSA level > 100 ng/mL and nine (28%) of 32 patients with PSA level 50–100 ng/mL had bone metastases in the present study. The number of bone metastatic Pca patients with PSA level > 100 ng/mL was significantly greater than that of patients with PSA level 50–100 ng/mL (*P* < 0.01).

**Table 1:** Patient characteristics

		CAB with GnRH antagonist	CAB with GnRH agonist	
		*n* = 34	*n* = 50	*P*-value
Median age at diagnosis years (range)		75(50–88)	74 (55–85)	0.09†
≥ 75 years old (%)		19 (56)	22 (44)	0.28‡
Median pretreatment PSA level ng/mL (range)		176.4 (51.5–5076)	136.4 (51.9–5827)	0.82†
Pathological diagnosis	Gleason score 6	1	2	0.78‡
	Gleason score 7	9	8	
	Gleason score 8	11	21	
	Gleason score 9	11	16	
	Gleason score10	2	3	
No of Gleason score 8–10 (%)		24(70)	40(80)	0.19‡
T category	T2	4	9	0.60‡
	T3	24	33	
	T4	6	7	
	Unknown	0	1	
Bone metastasis	EOD0	16	22	0.72‡
	EOD1	7	9	
	EOD2	8	10	
	EOD3	3	6	
	EOD4	0	1	
	Unknown	0	2	
Lymph node metastasis (%)	Positive	21 (62)	25 (50)	0.58‡
	Negative	13 (38)	23 (46)	
	Unknown	0	2	
When to use bicalutamide	Concurrently	15	12	
	Before	19	38	
Median observation period days (range)		761 (212–1529)	1141(175–3419)	< 0.01*

†: Wilcoxon’s rank sum test, ‡: Chi-square test, *: Log-rank test

*CAB* combined androgen blockade, *EOD* the extent of disease, *GnRH* gonadotropin-releasing hormone, *PSA* prostate-specific antigen

EOD stratification

EOD grade 0; normal and benign bone disease

EOD grade 1; number of bone metastases < 6

EOD grade 2; number of bone metastases 6–20

EOD grade 3; number of bone metastases > 20 but less than “super scan”

EOD grade 4; super scan

There were no significant differences between the two groups in terms of age at the time of diagnosis, bone metastasis, lymph node metastasis, GS, or pretreatment PSA level. The two groups were not mismatched with regard to patient characteristics, but there were differences in observation period between the groups. No patients in either group suffered from cardiovascular disease during the observation period.

PSA recurrence within 1 year was observed in five patients (15%) in the CAB-antagonist group, and one patient died from Pca during the observation period. PSA recurred within 1 year in 22 patients (44%) in the CAB-agonist group. Twenty-one patients died from Pca among those patients with PSA recurrence in the CAB-agonist group during the observation period (Fig. 1). All patients with PSA recurrence had bone or lymph node metastasis at the time of diagnosis.

**Fig. 2:**
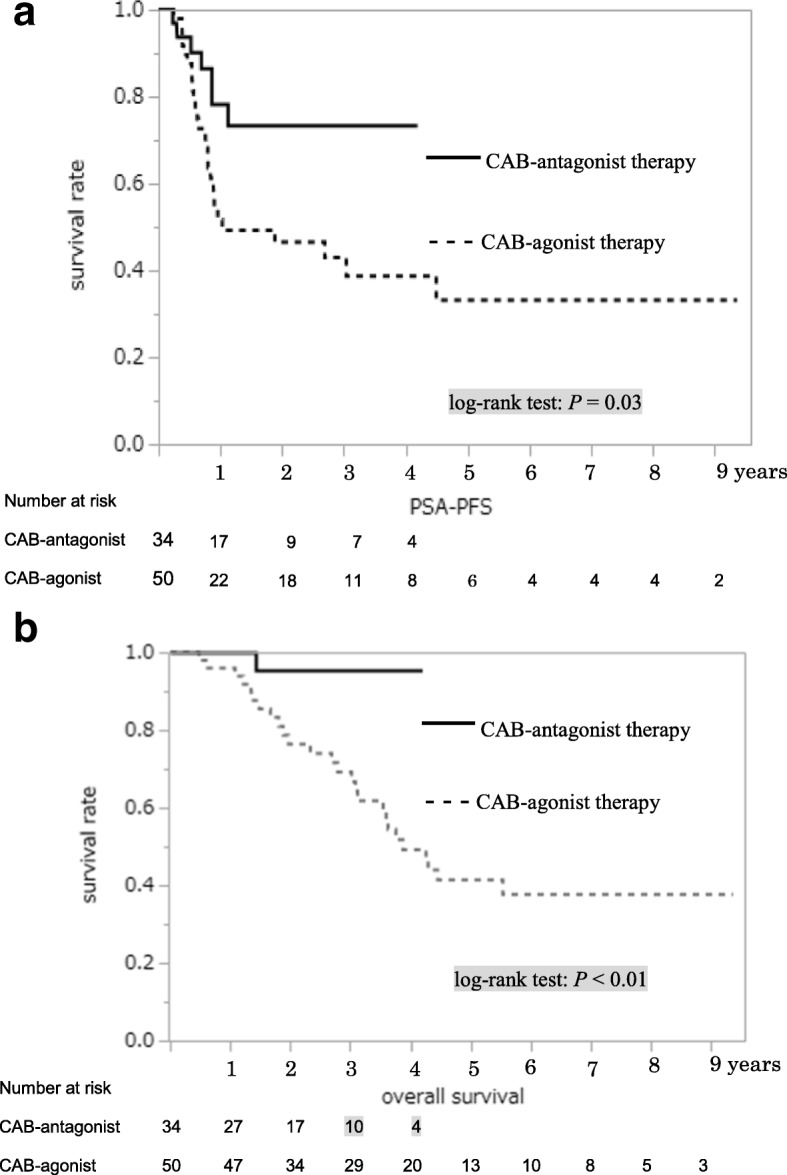
Kaplan–Meier curves of prostate-specific antigen progression-free survival (PSA-PFS) (**a**) and overall survival (OS) (**b**) showed significantly better results in the group undergoing combined androgen blockade therapy with concurrent gonadotropin-releasing hormone antagonist (CAB-antagonist therapy) than the group undergoing combined androgen blockade therapy with concurrent gonadotropin-releasing hormone agonist (CAB-agonist therapy) among prostate cancer patients with pretreatment PSA level ≥ 50 ng/mL

**Fig. 3:**
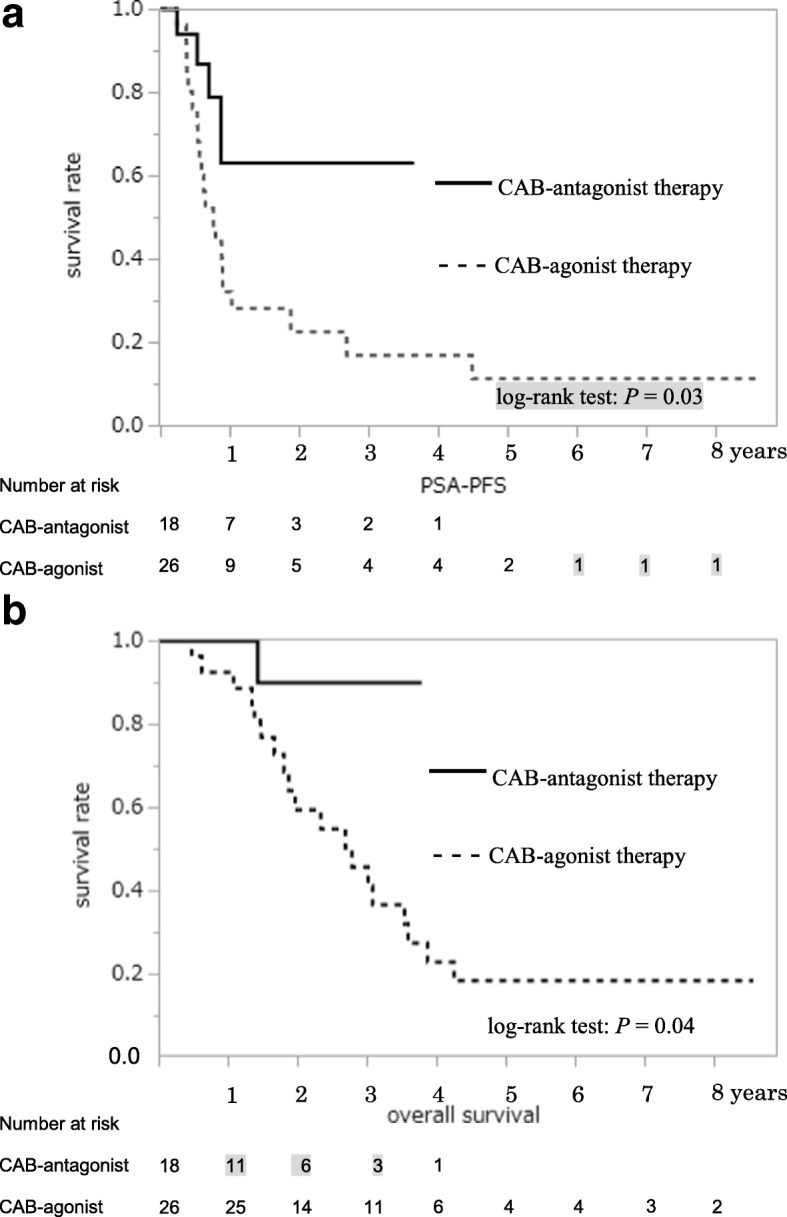
Kaplan–Meier curves for prostate-specific antigen progression-free survival (PSA-PFS) (**a**) and overall survival (OS) (**b**) showed significantly better results in the group undergoing combined androgen blockade therapy with concurrent gonadotropin-releasing hormone antagonist (CAB-antagonist therapy, *n* = 18) than the group undergoing combined androgen blockade therapy with concurrent gonadotropin-releasing hormone agonist (CAB-agonist therapy, *n* = 26) among patients with bone metastatic prostate cancer

**Fig. 4:**
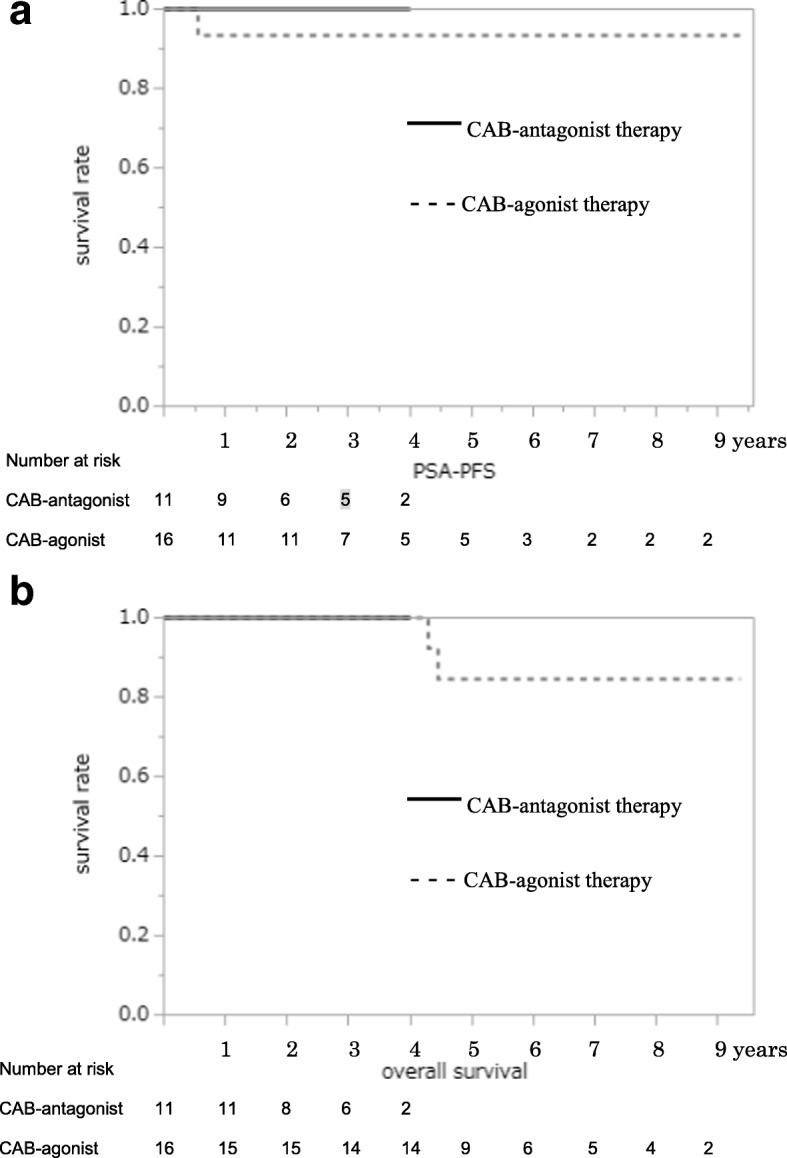
Kaplan–Meier curves for prostate-specific antigen progression-free survival (PSA-PFS) (**a**) and overall survival (OS) (**b**) showed no difference between the group undergoing combined androgen blockade therapy with concurrent gonadotropin-releasing hormone antagonist (CAB-antagonist therapy, *n* = 11) and the group undergoing combined androgen blockade therapy with concurrent gonadotropin-releasing hormone agonist (CAB-agonist therapy, *n* = 16) in patients without metastatic prostate cancer

PSA-PFS (Fig. 2a) and OS (Fig. 2b) of patients with PSA level ≥ 50 ng/mL were significantly different between the two groups in the Kaplan–Meier estimate. Among cases with bone metastasis, there were significant differences in PSA-PFS (Fig. 3a) and OS (Fig. 3b) between the two groups. Patients with PSA level ≥ 50 ng/mL without metastasis between both groups showed no differences in PSA-PFS (Fig. 4a) or OS (Fig. 4b). Eight of 27 patients without metastasis received adjuvant radiation therapy, three patients were in the CAB-antagonist group and five were in the CAB-agonist group.

**Fig. 5:**
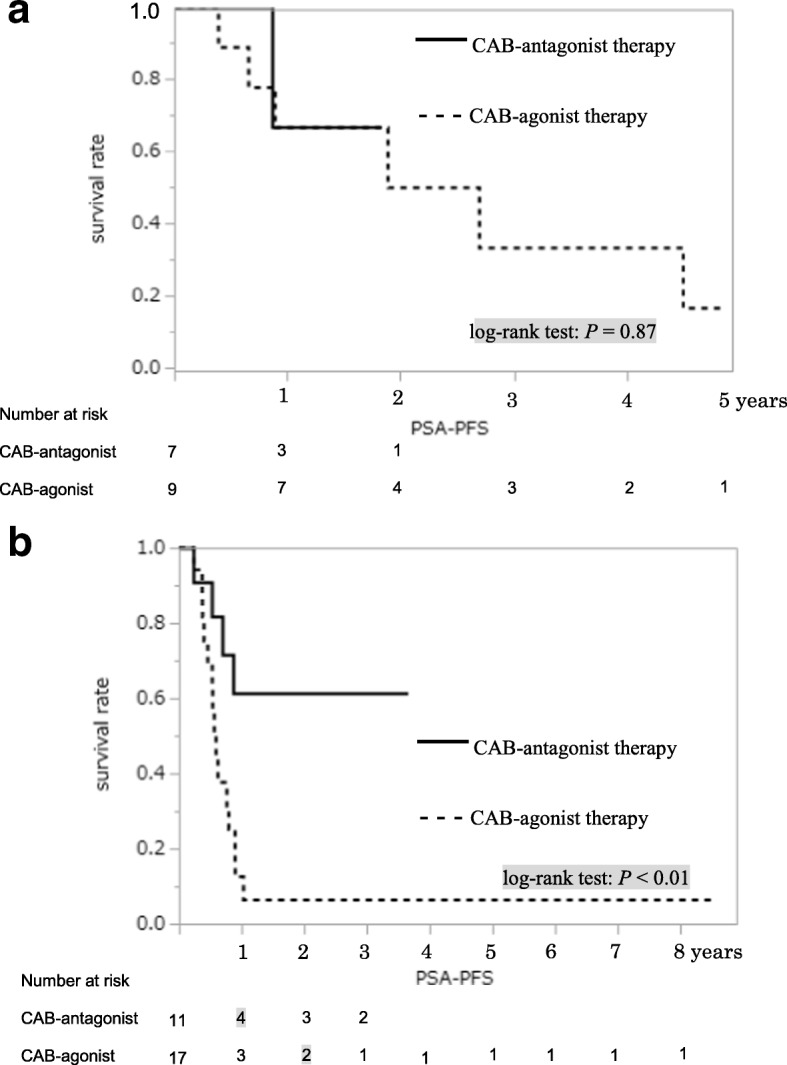
Kaplan–Meier curves for prostate-specific antigen progression-free survival (PSA-PFS) showed significantly better results in the group undergoing combined androgen blockade therapy with concurrent gonadotropin-releasing hormone antagonist (CAB-antagonist therapy, *n* = 11) than the group undergoing combined androgen blockade therapy with concurrent gonadotropin-releasing hormone agonist (CAB-agonist therapy, *n* = 17) in prostate cancer patients with more than six bone metastases (**b**). There were no differences between the two groups in prostate cancer patients with less than six bone metastases (**a**)

**Fig. 6:**
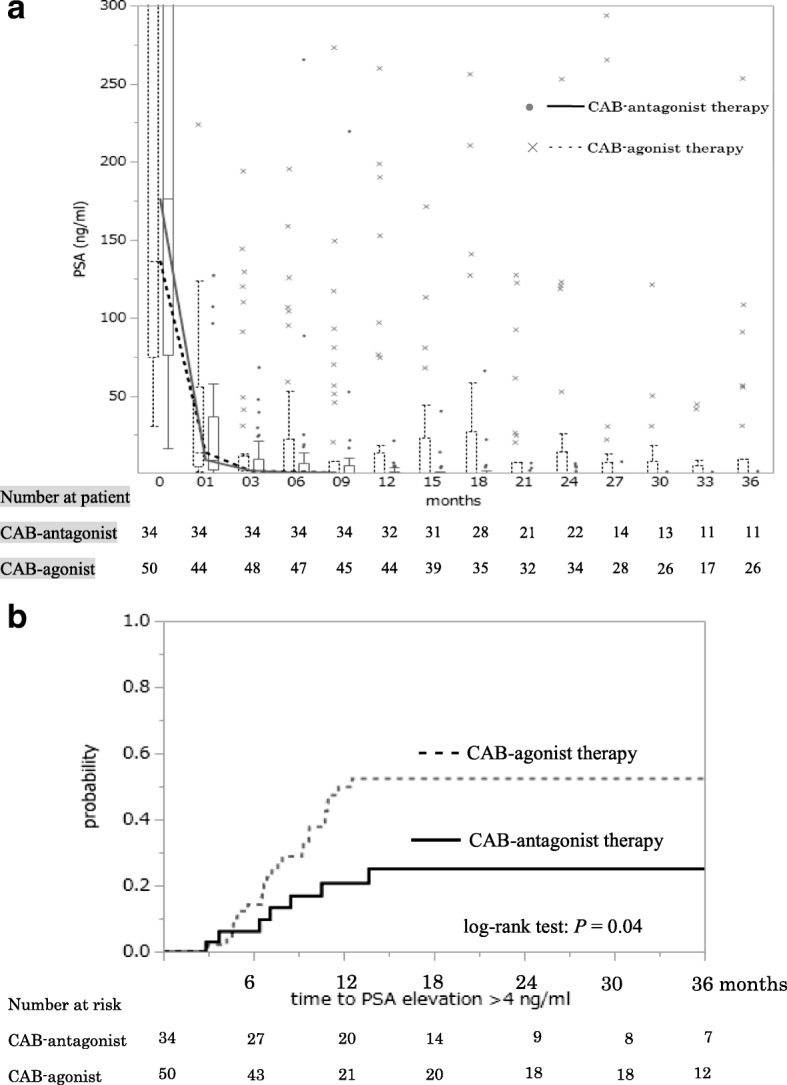
**a** Prostate-specific antigen (PSA) level during treatment in the two groups The black line shows the median PSA level in combined androgen blockade (CAB) therapy with concurrent gonadotropin-releasing hormone (GnRH) antagonist (CAB-antagonist therapy) and the black-dotted line shows the median PSA level in CAB therapy with concurrent GnRH agonist (CAB-agonist therapy). The box plot indicates upper whisker (+ 1.5 interquartile range), upper quartile, median, lower quartile, and lower whisker (− 1.5 interquartile range), respectively, with outlier marks. **b** The probability of PSA elevation > 4 ng/mL was significantly lower in CAB-antagonist therapy than CAB-agonist therapy in prostate cancer patients with PSA level > 50 ng/ml

The CAB-antagonist group with high volume disease, defined as more than six bone metastases (the extent of disease (EOD) grade 2–4), was associated with significantly greater improvement of PSA-PFS (Fig. 5b) in the Kaplan–Meier estimate compared to the CAB-agonist group [9, 10]. The two groups with low volume disease, defined as less than six bone metastases (EOD grade 1), showed no differences in PSA-PFS (Fig. 5a). Median PSA levels during treatment in the two groups are shown in Fig. 6a. There was a significant difference in PSA elevation > 4 ng/mL after commencement of CAB therapy between the two groups (Fig. 6b). On multivariate analysis, CAB-antagonist therapy was shown to be a significant prognostic factor for PSA-PFS (Table 2) in Pca patients with pretreatment PSA level ≥ 50 ng/mL.

**Table 2:** Multivariate analysis for PSA-PFS

	Univariate analysis			Multivariate analysis		
	HR	95% CI	*P*-value	HR	95% CI	*P*-value
≥ 75 years old at diagnosis	1.09	0.55–2.16	0.79	1.17	0.59–2.35	0.65
CAB therapy with GnRH antagonist	0.40	0.16–0.87	0.02	0.41	0.16–0.90	0.03
Bone metastasis positive	4.12	1.93–9.79	< 0.01	3.60	1.66–8.74	< 0.01
Gleason score ≥ 8	3.38	1.21–14.07	0.02	1.94	0.66–8.34	0.25

*CAB* combined androgen blockade, *CI* confidential interval, *HR* hazard ratio, *PFS* progression-free survival, *PSA* prostate-specific antigen

## Discussion

ADT has become the primary treatment option in cases of advanced Pca since Huggins et al. first reported the clinical efficacy of orchiectomy in such patients [11].

CAB therapy to block adrenal gland-derived testosterone was first reported in 1979. There have since been a number of reports that CAB therapy has a greater effect in improving survival rate than castration alone, but it has not been adopted as a standard therapy around the world [12, 13].

CAB therapy, in which a nonsteroidal antiandrogen is used along with GnRH agonist, has been used in 59% of patients with advanced Pca in Japan. CAB therapy is increasingly used with increasing Gleason score and clinical stage [14, 15]. In Japan, CAB therapy has been adopted as a standard treatment modality for advanced Pca. However, CAB-agonist therapy does not sufficiently improve prognosis in cases of bone metastatic Pca. Recently, next-generation CAB therapy with abiraterone and ADT was shown to significantly prolong OS and progression-free survival in metastatic Pca and HSPC compared to ADT alone. GnRH agonists as ADT were used for most metastatic Pca patients in these studies [3, 4]. The present study examined therapeutic effects of CAB therapy with GnRH antagonist and GnRH agonist concurrently using the conventional antiandrogen, bicalutamide in Pca patients with PSA level > 50 ng/mL.

GnRH antagonists competitively inhibit GnRH from the hypothalamus. These interactions result in suppression of luteinizing hormone (LH) and follicle-stimulating hormone (FSH) secretion from the pituitary gland. GnRH antagonists do not lead to a transient rise in testosterone (testosterone surge) as seen with GnRH agonists. After 1 month of treatment, 59% of patients treated with the GnRH antagonist, degarelix, reached a PSA level of ≤ 4 ng/mL, suggesting a faster pace of decrease in PSA level compared to patients treated with leuprorelin [6]. In the present study, the PSA level from baseline decreased rapidly in the two groups, however there was a significant difference in PSA elevation > 4 ng/mL after commencement of CAB therapy between the two groups. Metastatic Pca patients with PSA level > 4 ng/mL at 7 months after commencement of therapy could be early treatment failure and PSA levels rarely decreased further after 6 months [16]. PSA–PFS was associated with OS. [17]. PSA elevation > 4 ng/ml was clinically important to detect treatment failure at an early time point after commencement of primary CAB therapy. These findings indicated the efficacy of CAB-antagonist therapy for metastatic Pca.

In metastatic Pca, PSA recurrence was reported 18–24 months after the start of ADT. In the present study, PSA recurrence was observed at rates of 79 and 91% within 1 and 2 years, respectively. The PSA recurrence rate increased rapidly, particularly with PSA ≥ 50 ng/mL at the time of diagnosis. Particularly for clinical cases with pretreatment PSA ≥ 50 ng/mL, the PSA recurrence rate within 1 year decreased to 29% for the degarelix group, in comparison to 40% for the leuprorelin group [6]. In the present study, the PSA recurrence rates within 1 year were 15% for the CAB-antagonist group and 44% for the CAB-agonist group. Thus, CAB-antagonist group showed a significantly reduced PSA recurrence rate, suggesting that the PSA recurrence rate using CAB-antagonist may be lower than that with GnRH antagonist alone.

The results of the present study indicated that CAB-antagonist therapy improved PSA-PFS to a significantly greater extent than CAB-agonist therapy. This suggests that CAB therapy using both GnRH antagonist and nonsteroidal antiandrogen agent may prevent PSA recurrence and further improve PSA-PFS. In advanced Pca, the retention of PSA at a low level by initial-phase endocrine therapy improves the prognosis [16]. It was reported that GnRH antagonist significantly prolonged PSA-PFS compared to treatment with GnRH agonist [18]. In cases of metastatic Pca, CAB-agonist therapy significantly improved OS in cases with lymph node metastasis. In contrast, CAB-agonist therapy did not improve OS in cases with bone metastasis. To improve the prognosis of advanced Pca, it is necessary to improve the prognosis of bone metastatic Pca. Our findings indicated that CAB-antagonist therapy prolonged PSA-PFS to a greater extent than CAB-agonist therapy in Kaplan–Meier estimate. The greater improvement of PSA-PFS by CAB-antagonist therapy may be clinically significant for metastatic Pca.

Currently abiraterone or docetaxel with ADT have been reported to be beneficial in cases of high-volume disease stratified according to visceral metastasis or the number of bone metastases [3, 4, 10]. In the present study, CAB-antagonist therapy with more than six bone metastases (EOD grade 2–4) was associated with greater improvement in PSA-PFS than CAB-agonist therapy. Multivariate analysis indicated that CAB-antagonist therapy was a possible prognostic factor for PSA-PFS.

Although the reasons for the above observations are not yet clear, there are a number of possible underlying mechanisms. One mechanism may involve the lack of testosterone surge when degarelix is used, as the flare phenomenon defined as aggravation of Pca is stimulated by the testosterone surge, resulting in death in some cases [19]. In cases with advanced and metastatic Pca, including those with pretreatment PSA ≥ 50 ng/mL, antiandrogen therapy is used before GnRH agonist to prevent the flare phenomenon. However, it has been reported that the testosterone surge occurs in 74% of patients even with antiandrogen administration [20]. In cases with pretreatment PSA ≥ 50 ng/mL, PSA-PFS showed a more significant improvement in the group treated with degarelix alone than in the group given GnRH agonist, in which antiandrogens were administered to prevent the flare phenomenon [8]. Therefore, degarelix, which is not associated with a testosterone surge, may improve the prognosis of metastatic Pca.

Another mechanism involves the administration of degarelix to inhibit FSH secretion. In recent years, attention has focused on the influence of FSH in Pca, which is as significant as that of testosterone [21]. Degarelix was reported to be capable of maintaining FSH at a low level during the period of treatment [20].

FSH is considered important for bone metabolism as it regulates the control of bone resorption and the formation of osteoblasts and osteoclasts. Degarelix maintains alkaline phosphatase (ALP), a marker of bone metastasis, at a low level during the period of treatment. ALP level rose in the GnRH agonist group 10 months after commencement of treatment [7]. The delayed ALP rise after commencement of GnRH agonist administration may represent a therapeutic failure for advanced Pca. This phenomenon suggests that the impacts of the testosterone surge and the transient rise in FSH in the initial phase of GnRH agonist treatment remain intact even after inhibition of testosterone. The lack of a delayed increase in ALP after commencement of degarelix treatment suggests that the aggravation of bone metastasis is inhibited. FSH receptor expression has been confirmed in cases of castration-resistant prostate cancer (CRPC). Lower FSH level is associated with longer period of progression from HSPC to CRPC [22]. The observations with degarelix treatment suggested that delayed transition to CRPC may be related to improved prognosis of bone metastatic Pca.

There have been few reports regarding the effectiveness of CAB-antagonist therapy as the initial-phase endocrine therapy in metastatic Pca. The results of the present study suggest that CAB-antagonist therapy may reduce PSA recurrence and prolong PSA-PFS in bone metastatic Pca. Thus, the present study suggested that CAB-antagonist therapy may improve the prognosis of bone metastatic Pca with EOD grade 2–4.

The survival of Pca patients is related to several risk factors, including the extent of the tumor, pathological grade, patient’s age, and pretreatment PSA level [23–26]. Patients with lymph node metastases were reported to have better outcome than those with bone metastases [26]. Pretreatment PSA levels could be associated with bone metastases in the present study. Prognostic factors consisting of patient’s age at the time of diagnosis, bone metastasis, GS, and application of CAB-antagonist therapy would be suitable for the present multivariate analysis of PSA-PFS. CAB-antagonist therapy was found to be a possible prognostic factor for PSA-PFS; however, the frequency of PSA recurrence and number of deaths were so small that there may have been confounding factors or bias that were not addressed in the present study.

The limitations of the present study include the small number of the two groups, the study was performed in a single institute, its retrospective nature, concern regarding matching between the two groups in terms of the patient population with Gleason score 8–10. Risk of bias resulting from differences in number at risk in each year on the Kaplan–Meier curve for PSA-PFS must be taken into consideration, because the observation periods between the two groups were significantly different and would represent an important source of bias. Therefore, the present study may not have allowed adequate assessment of the effects of CAB-antagonist therapy for bone metastatic prostate cancer. Despite these limitations, our findings could help to improve the prognosis of bone metastatic prostate cancer patients. Further large-scale prospective studies with well-matched groups of patients are required to confirm our findings.

## Conclusions

In cases of bone metastatic Pca with pretreatment PSA level ≥ 50 ng/mL, CAB-antagonist therapy as the primary ADT could be associated with greater prolongation of PSA-PFS than CAB-agonist therapy. CAB-antagonist therapy may be a useful therapeutically option for treatment of bone metastatic Pca patients with EOD grade 2–4.

## Abbreviations

ADT: Androgen deprivation therapy
ALP: Alkaline phosphatase
CAB: Combined androgen blockade
CAB-agonist: Combined androgen blockade with concurrent use of gonadotropin-releasing hormone agonist
CAB-antagonist: Combined androgen blockade with concurrent use of gonadotropin-releasing hormone antagonist
CI: Confidence interval
CRPC: Castration-resistant prostate cancer
EOD: The extent of disease
FSH: Follicle-stimulating hormone
GnRH: Gonadotropin-releasing hormone
GS: Gleason score
HR: Hazard ratio
HSPC: Hormone-sensitive prostate cancer
LH: Luteinizing hormone
OS: Overall survival
PFS: Progression-free survival
PSA: Prostate-specific antigen
PSA-PFS: Prostate-specific antigen progression-free survival
